# Accelerating computational discovery of porous solids through improved navigation of energy-structure-function maps

**DOI:** 10.1126/sciadv.abi4763

**Published:** 2021-08-13

**Authors:** Edward O. Pyzer-Knapp, Linjiang Chen, Graeme M. Day, Andrew I. Cooper

**Affiliations:** 1 IBM Research Europe, Hartree Centre, Sci-Tech Daresbury, Warrington, UK.; 2 Leverhulme Research Centre for Functional Materials Design, Department of Chemistry and Materials Innovation Factory, University of Liverpool, Liverpool, UK.; 3 School of Chemistry, University of Southampton, Southampton, UK.

## Abstract

While energy-structure-function (ESF) maps are a powerful new tool for in silico materials design, the cost of acquiring an ESF map for many properties is too high for routine integration into high-throughput virtual screening workflows. Here, we propose the next evolution of the ESF map. This uses parallel Bayesian optimization to selectively acquire energy and property data, generating the same levels of insight at a fraction of the computational cost. We use this approach to obtain a two orders of magnitude speedup on an ESF study that focused on the discovery of molecular crystals for methane capture, saving more than 500,000 central processing unit hours from the original protocol. By accelerating the acquisition of insight from ESF maps, we pave the way for the use of these maps in automated ultrahigh-throughput screening pipelines by greatly reducing the opportunity risk associated with the choice of system to calculate.

## Teaser

In silico design of materials is accelerated by 100× using AI to intelligently select which simulations to run.

## INTRODUCTION

In principle, the combination of machine learning and virtual computational screening is a powerful method for the discovery of new functional organic materials (*1*, *2*). Computational techniques show great promise for the calculation of both the thermodynamic stability and the associated functional properties of candidate materials, but it is difficult in practice to exploit these methods across a broad range of problems. A central challenge is the prohibitive computational expense of accurately calculating energies and properties for every candidate material that is to be screened, and machine learning may provide notable benefit here.

One of the most challenging cases is the a priori design of functional molecular organic crystals with desirable materials properties. Unlike their framework-based counterparts, such as zeolites and Metal Organic Frameworks (MOFs) (*3*–*5*), molecular crystals rarely obey simple geometric principles that can be exploited for rational design. Even very small changes to molecular structure can have marked effects on crystal packing and, hence, the resultant solid-state properties. Molecular crystal packing is often dictated by weak, competing intermolecular interactions: Hence, the a priori design of materials with predetermined, desirable properties requires a more subtle approach than for materials where structure (and hence function) can be “built-in” through the use of intuitive bonding rules, such as adherence to known framework topologies or other geometric bonding principles.

### Energy-structure-function maps

Energy-structure-function (ESF) maps are a combination of crystal structure prediction (CSP) with per-structure property calculation, which has been shown to be a powerful tool for the virtual screening of candidate organic molecules for desirable properties such as natural gas storage capacity (*6*) and charge carrier mobility (*7*). In an ESF map, candidate crystal structures are generated using CSP methodologies, which are then screened virtually for a desired property. The resulting pairing of lattice energy and function is then used as an indicative tool for the propensity of the molecule to express the desired properties. This information can be used to guide an experimental campaign, which has been used to validate this ESF map approach (*6*, *8*). However, while this strategy can be effective, generation of the ESF map can be computationally intensive. For example, for methane storage predictions (*6*), it took around 800,000 central processing unit (CPU) hours to compute an ESF map for only one of the molecules in the study (T2E), and this computational cost was distributed roughly equally between the CSP and property calculations. The cost of computing ESF maps grows as the property of interest becomes more computationally expensive and also when the ESF maps contain larger numbers of candidate structures; this is particularly problematic for porous materials, where the energy range that includes all observable crystal structures is extended by solvent templating. Multiple components (e.g., cocrystals) and multiple stable molecular conformers also increase the dimensionality of the energy landscape markedly (*8*, *9*).

### Bayesian optimization

Bayesian optimization (*10*) is a technique for evaluating a so-called black box function; that is to say, a function for which there is not access to the analytical, closed form

$${\text{min}}_{x\in Χ}f(x)$$

Bayesian optimization has become popular recently in the machine learning community for the efficient tuning of the hyperparameters of deep learning models (*11*), but given its strengths as a global optimizer and its powerful theoretical guarantees (*12*), it has also started to find applications in a more diverse set of domains (*13*–*16*). The core application area of Bayesian optimization is when each sample of the function, *f*, is expensive to acquire in financial cost, acquisition time, or both. This makes this approach highly attractive for our goal of more efficiently navigating large ESF maps.

Bayesian optimization has two fundamental principles. First, it promotes the use of a surrogate function, $\hat{f}$, to represent the true (unknown) function, *f*, that is being optimized. Since each data point is likely to be expensive to acquire, it is important that this surrogate function has robust and well-defined uncertainties associated with its evaluation. In this study, this model is a Gaussian process (*17*), although other models have been used (*18*, *19*).

A Gaussian process is a nonparametric machine learning model, which can be described by a Normal distribution N with mean function, μ, and a kernel function, *K*(*x*, *x*′)

p(f∣X)=N(μ,K(x,x′))

where *p*(***f***∣*X*) is the probability of ***f*** given *X*, and ***f*** is the vector of function values [*f*(*x*_1_), *f*(*x*_2_)…*f*(*x_N_*)] evaluated at input points *x*_1,_, *x*_2_…*x_N_*. There are many potential choices for the kernel function *K*(*x*, *x*′) and, for this study, we used a Matérn kernel (*20*)

$$C_{\frac{3}{2}}=\sigma^{2}(1+\frac{\sqrt{3}}{l})\text{exp}(-\frac{\sqrt{3}}{l})$$

where the length scale *l* is determined on a per-feature basis using the automatic relevance determination (*21*) protocol and σ^2^ is the signal SD. We also introduce a white noise kernel, whose scale is determined as a hyperparameter of the overall Gaussian process and tuned to maximize the log-likelihood of the model with respect to the data.

The second major principle of Bayesian optimization is to balance exploration (the acquisition of new knowledge) and exploitation (the reliance on existing knowledge) when deciding which data points to acquire (*22*). This takes advantage of the existence of the uncertainties associated with the evaluations of the surrogate function, $\hat{f}$, and is controlled through a construct known as the acquisition function. There are a number of potential acquisition functions, with the most popular being expected improvement (EI) (*23*), which aims to maximize the EI to the optimization of collecting a data point. While EI is seemingly a serial methodology, there have been strategies implemented recently that generalize to the parallel setting (*18*, *24*–*27*). Typically, these do not scale well with the number of dimensions and those which do require sparsity and incoherence properties of the feature space that are not present in this problem (*26*). Thompson sampling (*28*) solves this problem by approximating the predictive distribution as follows

$$p(y_{j}|x_{j},D_{T})=\int p(y_{j}|x_{j},\theta )p(\theta ,D_{T})d\theta$$

where *p*(θ) represents the prior distribution given a set of data $D_{T}$, thus approximating the posterior distribution using Monte Carlo, based on a single sample from $p(\theta ,D_{T})$. This method thus scales significantly better with the scale and dimensionality of the problem.

**Fig. 1:**
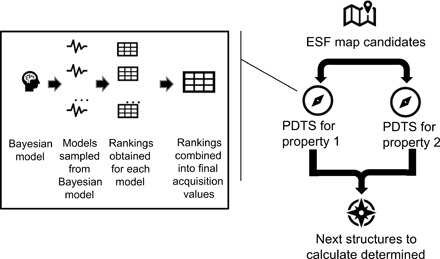
Graphical illustration of the Bayesian optimization framework used in this study.

The use of Thompson sampling for parallel Bayesian optimization requires an adaptation of this methodology known as parallel and distributed Thompson sampling (PDTS) (*18*), which is described visually in the inset of Fig. 1 and extensively in pseudo-code in the electrospray ionization. PDTS extends the Thompson sampling framework to a parallel case, exploiting the fact that PDTS with batch size *S* is the same as running sequential Thompson sampling *S* times without updating the current posterior. This allows the parallel and distributed calculation of the acquisition function, ensuring that this method is highly scalable with increasing batch size. This is particularly important in the case described here since it allows for evaluations to be distributed over a cluster computer system or even over a completely distributed system, such as IBM’s World Community Grid (*29*), which harnesses the power of volunteer compute by harvesting “idle” cycles from volunteer devices such as laptops, small computational systems, or even mobile devices.

In this study, we further extend the use of PDTS to the multiobjective case (MO-PDTS) without harming the scalability and thus the parallel performance. To achieve this, we assign a separate PDTS sampler to each objective, the acquisition functions of which are then combined in a single step, determining the final acquisition function for the overall optimization process. Under a Gaussian process prior, this combination is equivalent to optimizing a single objective consisting of a weighted combination of objective values, with one significant advantage. Since the acquisition values are distinct from the models used to predict them in our MO-PDTS setting, each sampler can be built from a completely different set of descriptors. Under the reasonable assumption that a model built from specifically chosen descriptors is more likely to have strong predictive ability than one built from a general set of descriptors, the ability to separate the predictors affords the user a framework that is significantly more transferable across a range of property types. The ability to fully distribute this calculation is maintained because the domain over which this optimization is performed is a discrete set of structures.

## RESULTS AND DISCUSSION

**Fig. 2:**
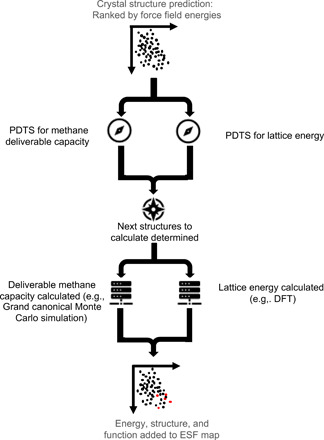
Flowchart representing the use of MO-PDTS for accelerating ESF map construction. Note that, in some cases, a sufficiently accurate value for lattice energy is calculated at the initial generation stage, and, in these cases, calculation of lattice energy is not necessary, but a second, optional calculation at a higher level may also be used.

**Fig. 3:**
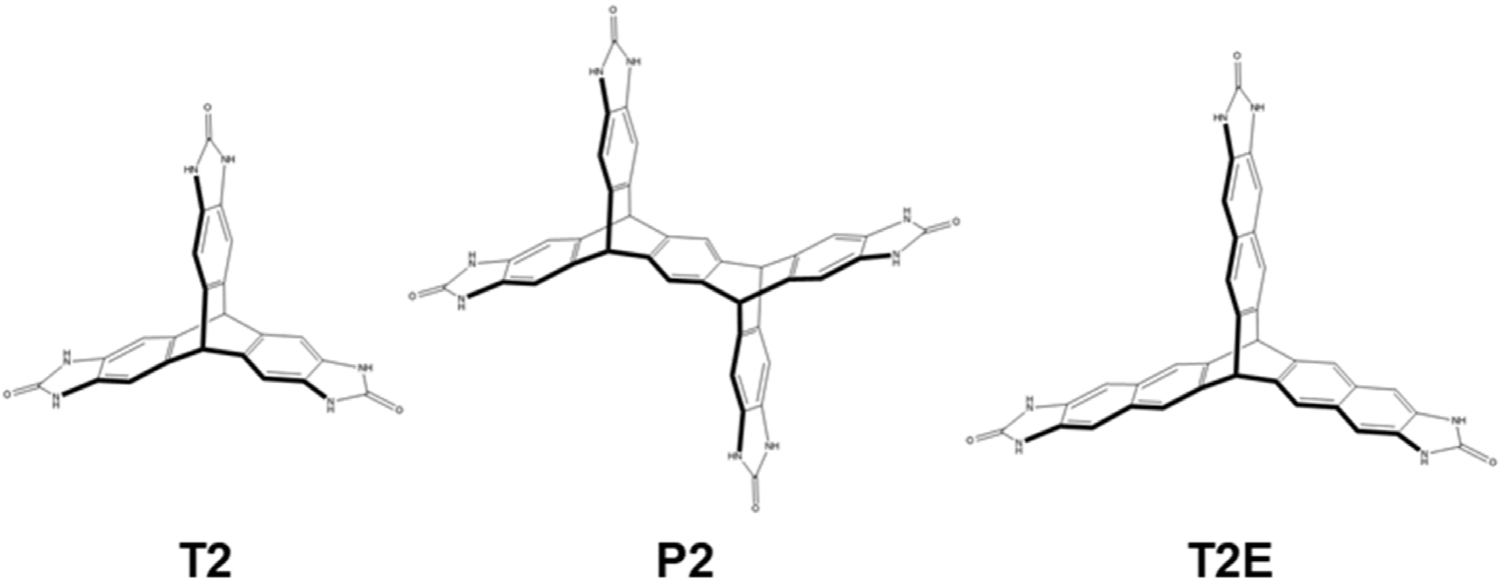
Chemical structures of the three molecules in this study.

We investigated the extent of MO-PDTS acceleration on three ESF maps that were calculated to evaluate the potential of three molecular materials for methane storage and delivery. We demonstrate how this new navigation workflow (Fig. 2) would have reduced the necessary computation and resulting time to insight for three systems, i.e., T2, T2E, and P2 (Fig. 3), recently predicted to have stable crystal structures with desirable methane deliverable capacities. These molecules were originally chosen because they represent a set of awkwardly shaped molecules. Hence, they have the potential to form porous structures with high methane capacities, but intuitive packing arguments alone cannot provide sufficient insight to make a priori arguments about the relative potential of these three molecules to perform well in this application. Even if we could predict crystal packing intuitively, the methane deliverable capacity does not scale in a simple way with crystal density; hence, both lattice energy and function must be computed. ESF maps for this application are very expensive because of the large energy range of viable predicted crystal structures, taking into account the effects of solvent stabilization coupled with the high cost of the methane adsorption calculations. In the study of T2, P2, and T2E (Fig. 3), crystal structures in the range up to 100 kJ/mol above the global minimum were considered, as compared to a more usual energy range of 10 or 15 kJ/mol for crystal structure landscapes for nonporous packings. Since the number of structures on the landscape increases rapidly as we move away from the global minimum, this 7- to 10-fold increased energy range leads to a much larger concomitant increase in the number of crystal structures that must be considered.

The ESF maps for methane deliverable capacity for T2, P2, and T2E contained ~5400, ~9800, and ~ 30,000 structures, respectively. To ensure reproducibility and to display the robustness of our approach, we tested the intelligent navigation workflow for each system with 10 replicate experiments, each of which was seeded with different initial structures chosen from the landscape. Using these replicate experiments, we were able to use the bootstrap methodology to calculate confidence intervals for the convergence of each of the three systems with respect to ideal behavior. All of the samplers converged on an ideal solution before 100 samples, or 10 epochs, have been completed. Since the executions are completed in parallel, when we calculate the first encounter time, we must only base this evaluation on the epoch in which the global minimum was found; that is, there is no advantage to being found halfway through a batch.

Table 1 shows the distribution of performance over the 10 repeats with the best performance being achieved by the T2 system, which shows a mean first encounter time of 14.3 samples, or within two completed epochs. Both P2 and T2E have a mean first encounter time of around four epochs. This can be rationalized by considering the full ESF maps for these systems: There are more that have both a high methane deliverable capacity and a low lattice energy for T2 than for other systems, facilitating the discovery of high performing systems. T2 also exhibits superior performance in the magnitude of our normalized objectives, with a score of circa 1.6, as compared to P2’s score of circa 1.5 indicating that there is a more favorable trade-off between low energy and high methane capacity structures.

**Table 1:** Average performance achieved over 10 replicates for the three systems studied. Mean encounter time is the mean sample number at which the minimum is found, and mean epochs required is the sampling epoch in which this sample fell.

**Structure**	**Mean encounter** **time**	**Mean epochs** **required**
T2E	39.0	4
T2	14.3	2
P2	34.0	4

### Comparison to greedy sampling

An alternative approach to the reduction in computational cost for the exploration of large ESF maps, or other compound libraries, is to use a greedy sampling method. For this class of search algorithm, a model is built from existing data and used to predict values for data that have not yet been acquired. At each epoch of sampling, the candidate that has the largest predicted value is selected—or the smallest value, for minimization purposes—and added to the training set, from which the model is then refitted. Most traditional Qualitative Structure Property Relationship ( QSPR) methods use this methodology, either implicitly or explicitly, for accelerated materials discovery.

**Fig. 4:**
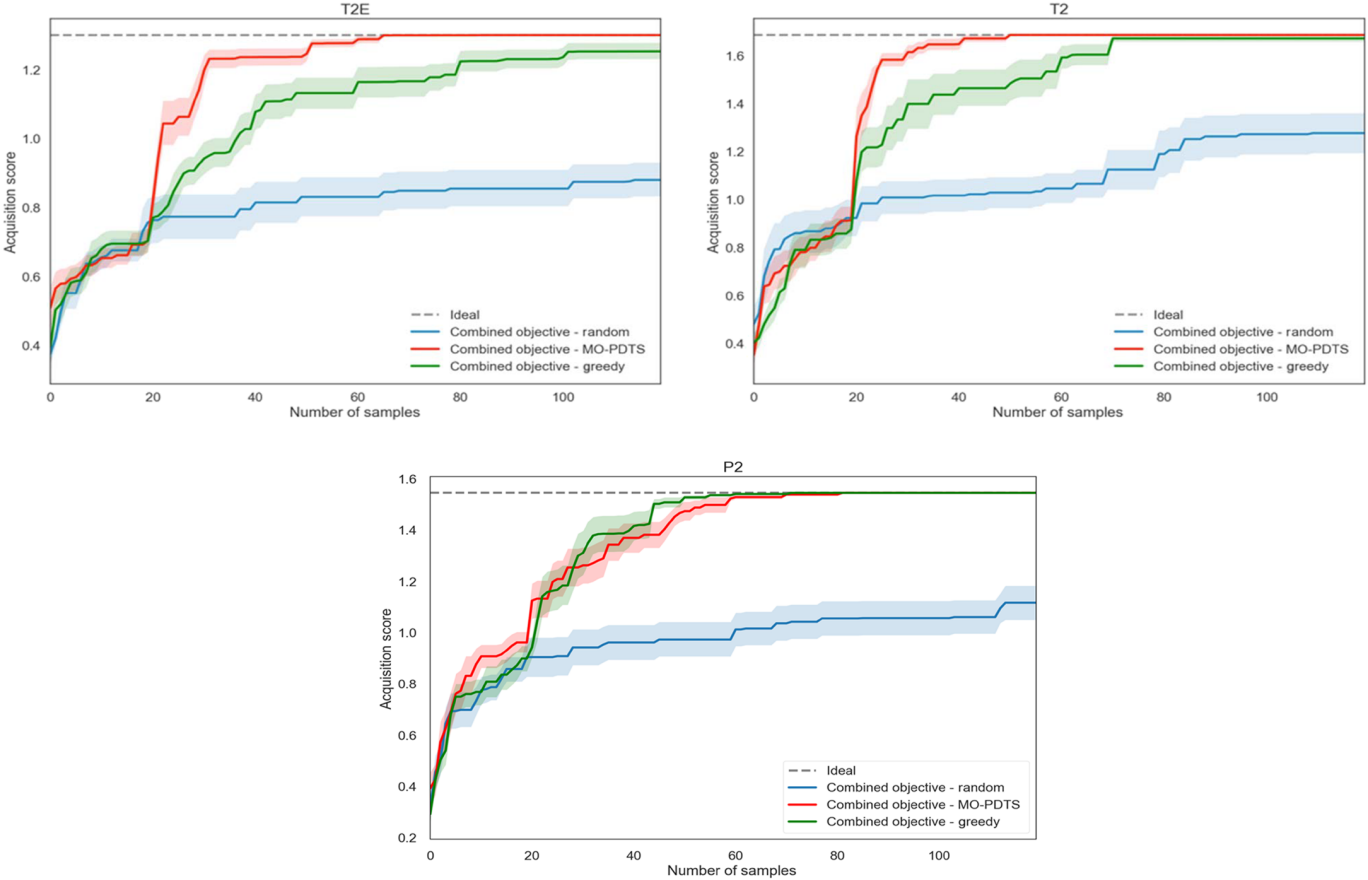
Performance of the MO-PDTS sampler for the three systems studied. Confidence intervals are generated using the bootstrap methodology from 10 replicate experiments seeded with different candidate structures.

As shown in Fig. 4, the MO-PDTS sampler locates the ideal solution in all cases and outperforms the baseline random sampler significantly. For T2E and T2 systems, there is a clear advantage over the greedy sampler, indicating that these are systems where there are competing local maxima and demonstrating the advantage of the more sophisticated MO-PDTS method. In the case of P2, the performances are similar, indicating that there is a single clear structure-property relationship, which can be exploited by the greedy sampler.

**Fig. 5:**
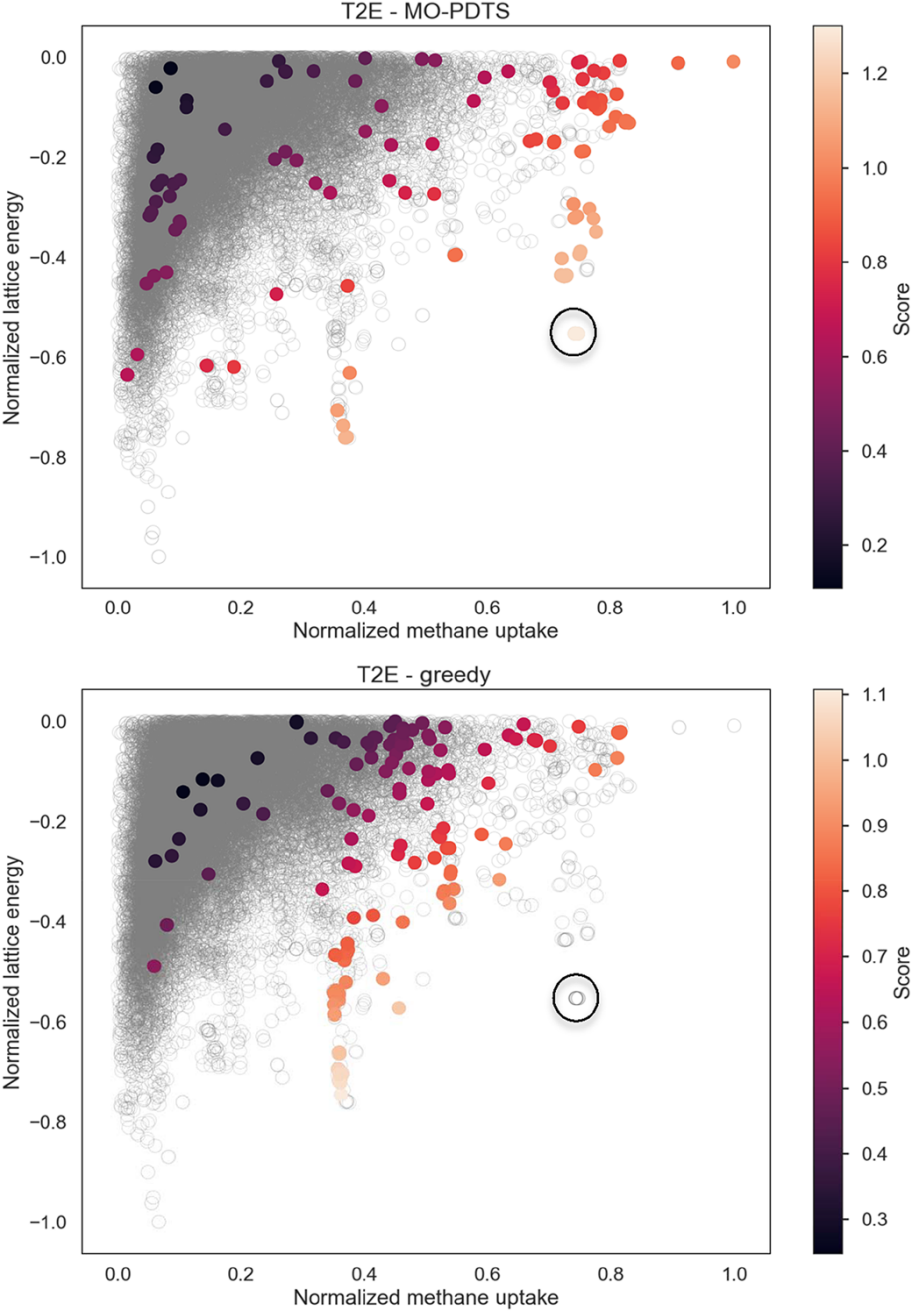
Comparison of the MO-PDTS and greedy sampling strategies. Candidate structures are colored by their combined energy-structure score, and no color indicates that the structure was not sampled. It can be seen, for the T2E case, that the greedy sampler gets stuck in a local maxima but that PDTS is able to locate the global maximum (circled in black)

The dangers of a greedy sampler are illustrated in the case of T2E (Fig. 5). The greedy sampler identifies a reasonably well-performing structure-property relationship and concentrates its sampling in this area. Unfortunately, this structure-property relationship does not indicate the existence of a second “peak” of activity with a higher value. The balance of exploration and exploitation in MO-PDTS avoids this situation and samples in a more intelligent and robust manner. The performance curves in Fig. 4 indicate that there is little “cost” to adopting this more sophisticated strategy over a more traditional, greedy approach when the structure-property landscape is simple but significant benefits when it is not.

### Computational savings

We have seen that the proposed intelligent navigation approach to ESF maps yields considerable computational savings. The exact details are shown in Table 2. In all cases, we see greater than two orders of magnitude improvement in the “time to insight,” which results in hundreds of thousands of saved CPU hours.

**Table 2:** Computational savings as a fraction of the potential ESF map for the systems T2, T2E, and P2.

**Structure**	**Number of structures in** **ESF map**	**% of ESF sampled**	**CPU hours to generate full** **ESF map**	Computational saving* **(CPU hours)**
T2E	29,848	0.14	392,213	391,427
T2	5403	0.32	74,469	73,945
P2	9817	0.39	96,369	95,583

*Computational saving is based on averaging the cost of each calculation over the entire set.

In total, 544,955 hours were saved using this technique over the entire campaign; for context, this saving is similar in magnitude to a small grant on a supercomputer system. For many functional properties, this high level of computational acceleration could transform ESF maps from a proof-of-concept demonstration to an important, routine practical tool for in silico high-throughput screening, particularly for physical properties that are expensive to compute. We assessed these savings based solely on the savings in property evaluations, but where higher-level energies are required for lattice energy rankings, for example, by density functional theory (DFT), the savings would be even greater. Even when we only consider the property calculation savings, these benchmark figures suggest that this technique could allow a user to screen orders of magnitude more candidates for the same computational expense. As with all accelerations of this kind, there is not the same completeness guarantee that is possible by calculating the entire ESF map. However, we believe that this is more than compensated by the huge increase in throughput and the ability to evaluate a much broader range of candidate molecular structures. In many cases, the use of this technique may be the difference between an ESF map for a particular property being calculated and being deemed too expensive. This represents a significant practical advance in the ESF methodology, allowing us to tackle new functional properties that have hitherto been deemed impossible because of their high computational cost.

In conclusion, we present an important evolution in the ESF mapping technique for the a priori prediction of materials properties: a smart navigator for ESF maps based on MO-PDTS. The scalability of this method adds negligible overhead to the computation of the ESF map; by selectively sampling the map and only requiring the use of expensive function calculations for a fraction of the structures, we are able to make significant computational savings. For the three structures here studied, we were able to save more than half a million CPU hours. This has two key advantages. First, we significantly reduce the opportunity risk for the selection of systems for ESF map calculation; that is, did I choose the right molecule to spend this resource on? Second, through the reduction of computational requirements, we extend the power of the ESF map approach both to researchers who are not able to access the necessary computational resources and also to expensive property calculations for large, complex ESF maps that are simply intractable today.

## MATERIALS AND METHODS

### MO-PDTS optimization details

We posed the problem as a multiobjective optimization over both energy and methane deliverable capacity, thus searching for the ESF maps for low-energy, highly porous, crystalline forms. For the purposes of this study, we are testing the methodology as if we do not have the final energies that we require, mimicking the case where higher-level energy calculations are required than were used in the structure search itself. We use the calculated force field energies as a proxy for these higher-level energies. We did not consider the expense for these energy calculations when calculating savings, and so this study represents a lower bound on the potential for this method.

To demonstrate the modular nature of this approach, the two considered properties were modeled with different features—22 geometrically defined features for porosity, and the National Institute of Standards and Technology JARVIS (*30*) descriptor set for the lattice energy.

Topological analysis of the pore space within a crystal structure was performed using the void analysis tool zeo++ (*31*). The outputs from this analysis included the pore dimensionality [zero dimension (0D), 1D, 2D, or 3D], pore diameters, surface areas, and pore volumes. A probe radius of 1.70 Å was used in all calculations. A total of 22 pore descriptors were calculated for each of the predicted crystal structures. These 22 descriptors are simple extensions to four basic pore descriptors: crystal density, largest pore diameter, total surface area, and total pore volume. First, the total surface area and the total pore volume were decomposed into accessible and nonaccessible contributions. Second, to capture the heterogeneity of the pore geometry within a structure, we derived several descriptors based on the surface areas and pore volumes of individual channels and pockets. Last, the total surface area was also decomposed into elemental contributions. A description of each descriptor is as follows:

1) Crystal density (in grams per cubic centimeter);

2 to 4) Pore diameters (in angstrom): the largest included sphere (*D*_i_), the largest free sphere (*D*_f_), and the largest included sphere along the free sphere path (*D*_if_);

5 to 8) Accessible surface area (in square meters per gram), nonaccessible surface area (in square meters per gram), accessible volume (in cubic centimeters per gram), nonaccessible volume (in cubic centimeters per gram);

9 to 12) Absolute (in cubic centimeters per gram) and fraction (−) of probe-occupied accessible volume, absolute (in cubic centimeters per gram), and fraction (−) of probe-occupied nonaccessible volume;

13 to 16) Elemental surface areas (in square meters per gram), i.e., total (accessible + nonaccessible) surface area decomposed into individual contributions from the H, C, N, and O atoms;

17 to 22) Variants based on the surface areas and pore volumes of individual channels [accessible (acc)] and pockets [nonaccessible (nacc)] to capture, to some extent, the heterogeneity of the pore space within a crystal structure:

Average of the accessible surface areas divided by the corresponding accessible volumes for all individual channels

$$A_{\text{acc}}=\frac{1}{n}\sum_{n=1}^{n}\frac{S_{n,\text{acc}}}{V_{n,\text{acc}}}$$

Median of the accessible surface areas divided by the corresponding accessible volumes for all individual channels

$$M_{\text{acc}}=\text{Median}(\frac{S_{1,\text{acc}}}{V_{1,\text{acc}}},\frac{S_{2,\text{acc}}}{V_{2,\text{acc}}}\cdots \frac{S_{n,\text{acc}}}{V_{n,\text{acc}}})$$

Variance of the accessible surface areas divided by the corresponding accessible volumes for all individual channels

$${\sigma^{2}}_{\text{acc}}=\frac{1}{n}\sum_{n=1}^{n}{\left(\frac{S_{n,\text{acc}}}{V_{n,\text{acc}}}-A_{\text{acc}}\right)}^{2}$$

Average of the nonaccessible surface areas divided by the corresponding nonaccessible volumes for all individual pockets

$$A_{\text{nacc}}=\frac{1}{n}\sum_{n=1}^{n}\frac{S_{n,\text{nacc}}}{V_{n,\text{nacc}}}$$

Median of the nonaccessible surface areas divided by the corresponding nonaccessible volumes for all individual pockets

$$M_{\text{nacc}}=\text{Median}(\frac{S_{1,\text{nacc}}}{V_{1,\text{nacc}}},\frac{S_{2,\text{nacc}}}{V_{2,\text{nacc}}}\cdots \frac{S_{n,\text{nacc}}}{V_{n,\text{nacc}}})$$

Variance of the nonaccessible surface areas divided by the corresponding nonaccessible volumes for all individual pockets

$${\sigma^{2}}_{\text{nacc}}=\frac{1}{n}\sum_{n=1}^{n}{\left(\frac{S_{n,\text{nacc}}}{V_{n,\text{nacc}}}-A_{\text{nacc}}\right)}^{2}$$

where *n* is the number of channels or pockets, *S*_*n*,acc_ and *V*_*n*,acc_ are the accessible surface area and accessible volume for the *n*th channel, respectively; and *S*_*n*,nacc_ and *V*_*n*,nacc_ are the nonaccessible surface area and nonaccessible volume for the *n*th pocket, respectively.

Since JARVIS is a very high-dimensional set of features with significant information redundancy, we use a principle component analysis to reduce the number of features while retaining 99% of the variance. This resulted in the feature dimensions for the systems shown in Table 3.

**Table 3:** Dimensionality of JARVIS descriptors, once reduced using principal components analysis to retain 99% of original variance.

**System**	**Number of dimensions**
T2	45
P2	59
T2E	28

To quantify the acceleration achieved, we compare our results here to the calculation of full ESF maps, previously reported by some of the authors (*6*); that is, we accurately computed both lattice energies and methane deliverable capacities for all structures on the three associated ESF maps. Since ESF maps are used as indicators of the potential for a molecule to behave in a desirable way, we based our metric of success on the first encounter time for the global minimum on the ESF landscape; that is to say, the structure that has the best combination of low energy and high methane deliverable capacity. For this study, we weighted the contribution to this score from the energy term and the property term equally

$$S=aE_{i}+bP_{i}$$

Where *a* and *b* are weighting coefficients to energy and property, respectively, and, in this study, are equal and normalized to remove units and ensure that the scales of the two properties are comparable. We note that, for a more conservative approach, it is possible to weight the energy term more highly, that is, to increase the likelihood that the identified structure is thermodynamically accessible in the laboratory.

**Fig. 6:**
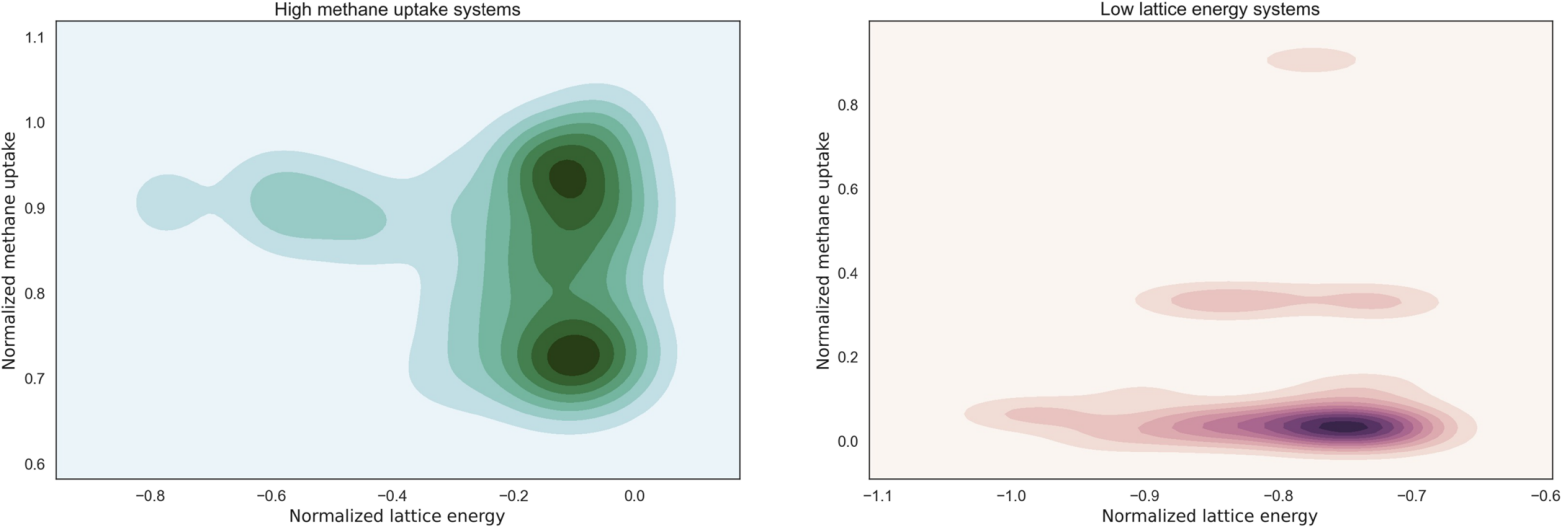
Estimated density plot for the normalized lattice energy and the normalized methane deliverable capacity for the T2 system, with values normalized over the entire dataset between 0 and 1 for methane capacity (−1 and 0 for energy). Color hue is used to indicate contour planes of increasing density. The left plot focuses on crystal packings with high methane deliverable capacity; the right plot highlights systems with low lattice energies. Most of the low energy systems have poor methane deliverable capacity, and the largest high methane capacity systems are relatively high in lattice energy; that is, these two properties are, broadly speaking, orthogonal.

Figure 6 shows that, in general, structures with high deliverable methane capacity have a high lattice energy. Thus, we expect that the number of structures, which have both desirable methane deliverable capacity and low lattice energy to be small, is further emphasizing the need for an efficient, accelerated approach and also the importance of the multiobjective nature of our search strategy.

The MO-PDTS was seeded with an initialization strategy based on *k*-means inspired by the generation of inducing points for sparse Gaussian processes. In this methodology, *k*-centroids were determined over input descriptor (feature) space using the *k*-means algorithm. The structures that minimized the distance to these centroids were chosen to initialize the search; that is, we selected the nearest structure to each of the *k*-centroids. Under a uniform distribution, this is equivalent to a Latin hypercube due to the spherical repulsion of *k*-means. However, under a nonuniform distribution, we believe that this initialization captures the underlying data structure better, leading to increased model stability throughout the optimization process. MO-PDTS was then run for 10 epochs, at each of which 10 structures were selected and properties were calculated. To account for the difference in magnitudes of the two objectives, the values for each were scaled for each objective based on the 20 selected structures from which the search was seeded.

### Simulation details

For each ESF map, candidate crystal structures were generated using a quasi-random sampling procedure, as implemented in the Global Lattice Energy Explorer software (*32*). Molecules were first sketched in ChemDraw, followed by an initial molecular geometry optimization with the COMPASS force field (*33*), as implemented in the Materials Studio software package (*34*). Force field–optimized molecular geometries were further refined by reoptimization using DFT with the M06-2X exchange-correlation functional and 6-311G** basis set. Molecular DFT calculations were performed with the Gaussian09 software (*35*). These molecular geometries were held rigid throughout crystal structure generation and lattice energy minimization.

Lattice energy calculations were performed with an anisotropic atom-atom potential using DMACRYS (*36*). Electrostatic interactions were modeled using an atomic multipole description of the molecular charge distribution (up to hexadecapole on all atoms) from the B3LYP/6-31G**-calculated charge density using a distributed multipole analysis (*37*). Atom-atom repulsion and dispersion interactions were modeled using a revised Williams intermolecular potential (*38*).

Methane adsorption was predicted for each structure at a temperature of 298 K and pressures of 5.8 and 65 bar; methane deliverable capacity was calculated as the difference in methane uptake at 65 and 5.8 bar (assuming gas storage at 65 bar and gas delivery at 5.8 bar). All of the adsorption predictions were performed using grand canonical Monte Carlo simulations involving a 50,000-cycle equilibration period and a 50,000-cycle production run, using the RASPA code (*39*). The adsorbent-adsorbate and adsorbate-adsorbate intermolecular interactions were modeled using Lennard-Jones (LJ) potentials, with a cutoff radius of 12.0 Å (beyond which a simple truncation was applied). Methane (CH_4_) was described by the TraPPE united-atom force field (*40*), in which CH_4_ is considered a single entity, i.e., the carbon atom and its bonded hydrogen atoms are grouped together to form one interaction site. The LJ parameters for the adsorbent structures were assigned on the basis of the DREIDING force field (*41*). The Lorentz-Berthelot combining rules were used to calculate the LJ cross-parameters.

## SUPPLEMENTARY MATERIALS

Supplementary material for this article is available at http://advances.sciencemag.org/cgi/content/full/7/33/eabi4763/DC1
